# Intensity standardization of MRI prior to radiomic feature extraction for artificial intelligence research in glioma—a systematic review

**DOI:** 10.1007/s00330-022-08807-2

**Published:** 2022-04-29

**Authors:** Kavi Fatania, Farah Mohamud, Anna Clark, Michael Nix, Susan C. Short, James O’Connor, Andrew F. Scarsbrook, Stuart Currie

**Affiliations:** 1 Department of Radiology, Leeds Teaching Hospitals NHS Trust, Leeds, UK; 2 Leeds Institute of Medical Research, University of Leeds, Leeds, UK; 3 Department of Radiology, Leeds General Infirmary, Great George Street, Leeds, LS1 3EX UK; 4 University of Leeds Medical School, Leeds, UK; 5 Department of Medical Physics, Leeds Teaching Hospitals NHS Trust, Leeds, UK; 6 Department of Clinical Oncology, Leeds Teaching Hospitals NHS Trust, Leeds, UK; 7 Division of Cancer Sciences, The University of Manchester, Manchester, UK; 8 Department of Radiology, The Christie Hospital, Manchester, UK; 9 Division of Radiotherapy and Imaging, Institute of Cancer Research, London, UK

**Keywords:** Magnetic resonance imaging, Glioma, Reproducibility of results

## Abstract

**Objectives:**

Radiomics is a promising avenue in non-invasive characterisation of diffuse glioma. Clinical translation is hampered by lack of reproducibility across centres and difficulty in standardising image intensity in MRI datasets. The study aim was to perform a systematic review of different methods of MRI intensity standardisation prior to radiomic feature extraction.

**Methods:**

MEDLINE, EMBASE, and SCOPUS were searched for articles meeting the following eligibility criteria: MRI radiomic studies where one method of intensity normalisation was compared with another or no normalisation, and original research concerning patients diagnosed with diffuse gliomas. Using PRISMA criteria, data were extracted from short-listed studies including number of patients, MRI sequences, validation status, radiomics software, method of segmentation, and intensity standardisation. QUADAS-2 was used for quality appraisal.

**Results:**

After duplicate removal, 741 results were returned from database and reference searches and, from these, 12 papers were eligible. Due to a lack of common pre-processing and different analyses, a narrative synthesis was sought. Three different intensity standardisation techniques have been studied: histogram matching (5/12), limiting or rescaling signal intensity (8/12), and deep learning (1/12)—only two papers compared different methods. From these studies, histogram matching produced the more reliable features compared to other methods of altering MRI signal intensity.

**Conclusion:**

Multiple methods of intensity standardisation have been described in the literature without clear consensus. Further research that directly compares different methods of intensity standardisation on glioma MRI datasets is required.

**Key Points:**

• *Intensity standardisation is a key pre-processing step in the development of robust radiomic signatures to evaluate diffuse glioma.*

• *A minority of studies compared the impact of two or more methods.*

• *Further research is required to directly compare multiple methods of MRI intensity standardisation on glioma datasets.*

**Supplementary Information:**

The online version contains supplementary material available at 10.1007/s00330-022-08807-2.

## Introduction

**Fig. 1:**
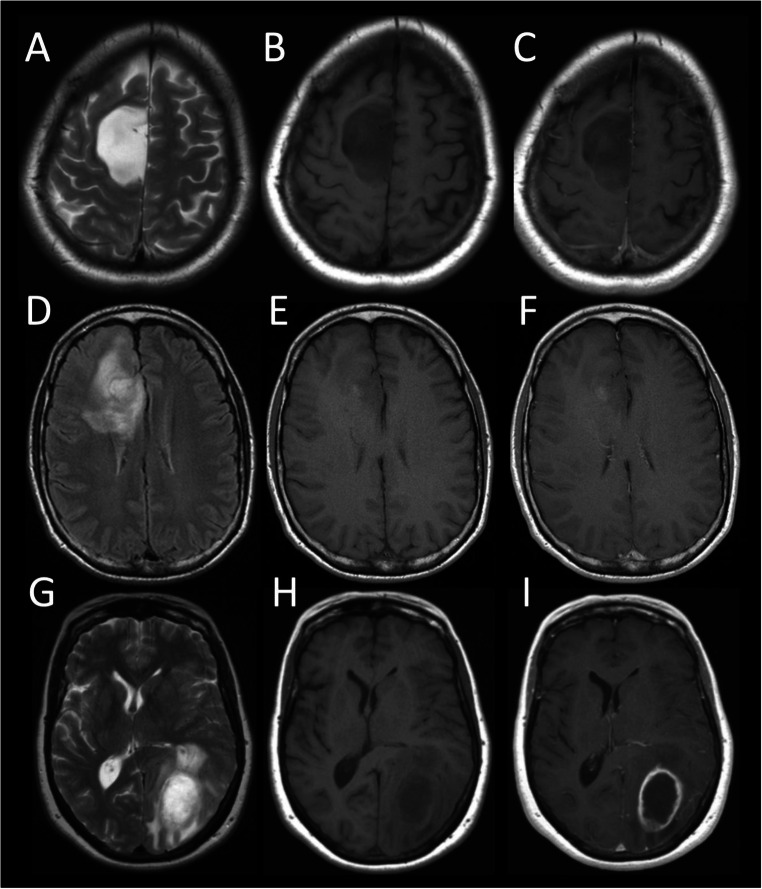
MR imaging in three different examples of adult-type diffuse gliomas

Adult-type diffuse gliomas are a varied group of highly invasive and heterogenous brain tumours (Fig. 1), with an annual US incidence of 5–6/100,000 and glioblastoma (GBM, the most aggressive glioma) accounting for nearly 50% [1]. Despite maximal safe resection of enhancing tumour, and adjuvant therapy with concomitant temozolomide chemotherapy and 60 Grey in 30 fractions of radiotherapy, followed by 6 cycles of temozolomide (‘Stupp protocol’), median overall survival of patients with GBM remains poor at 12–15 months [2, 3].

Multiparametric MRI (mpMRI), with its excellent soft tissue contrast, is frequently used to characterise these tumours [4]. Growing interest in using artificial intelligence (AI) to augment information provided by MRI includes, but is not limited to, non-invasive prediction of cytogenetic alterations, distinguishing treatment effects from pseudoprogression, and distinguishing infiltrative non-enhancing tumour from oedema [5].

Radiomics is a quantitative analytic method of extracting mineable data from medical imaging, and machine learning is typically used to correlate radiomic features and patient-specific data relating to prognosis and/or outcome [6]. Quantitative assessment of the whole tumour volume and surrounding tissues is attractive in the study of a heterogenous disease, which is hampering current treatment strategies [5]. Many radiomic studies evaluating types of diffuse glioma aim to predict prognosis [7], non-invasively diagnose genetic and molecular changes [8] (which play a key role in diagnosis, prognosis, and management), and distinguish between treatment effects and tumour progression [9].

Despite its promise, radiomics has largely been limited to small retrospective proof-of-principle studies, without sufficient evidence to support translation into radiological practice [10]. MRI-based radiomics is limited by the non-biological, scanner-dependent variation in image signal intensity [11–14]. MR intensity does not map easily to a physical tissue property, in contrast to CT, and shows variation between timepoints, vendors, magnetic field strengths, and acquisition settings [15–18]. Radiomic features are highly sensitive to the values of the signal intensities in the image, and non-biological alteration must be removed. Therefore, MRI signal intensity must be standardised, i.e. the range and distribution of voxel intensity must be similar across patients, prior to radiomic analysis to ensure that the results are reproducible [11]. Despite this, there is a lack of consensus as to the optimal method when characterising diffuse glioma. Although not a specific diagnosis, diffuse glioma is a useful grouping, as they often share the same radiomics pipeline and are a commonly studied group of related tumours [13, 16]. We aim to perform a systematic review of the literature examining the efficacy of different MRI intensity standardisation procedures prior to the extraction of radiomic features in the setting of adult-type diffuse glioma.

## Materials and methods

### Search strategy and selection criteria

This systematic review was undertaken according to the ‘Preferred Reporting Items for Systematic Reviews and Meta-Analysis’ (PRISMA) statement. A search of MEDLINE, EMBASE, and SCOPUS databases was performed on 5 October 2021 using the following concepts, linked by the “AND” operator, including synonymous terms that were linked with the “OR” operator: (1) MRI, (2) radiomics, (3) intensity standardisation, and (4) glioma. No limit was placed on the date, language, location, or type of study. Exclusion criteria were the following: non-human based, not regarding adult-type diffuse gliomas, non-original research, non-MR radiomics, no mention of intensity standardisation, or no assessment of the effect of intensity standardisation (compared to another method or to no standardisation). After removing duplicates, articles were screened based on titles and abstract, and subsequently the full text. References in the included articles were manually reviewed. Full search strategy, methodology, and PRISMA checklist are available in the supplementary files.

### Quality assessment

Quality Assessment of Diagnostic Accuracy Studies 2 (QUADAS-2) was used to assess the risk of bias [19]. QUADAS-2 was used because the objective was to evaluate performance of any given intensity standardisation method, when compared to either no standardisation or another method. QUADAS-2 assesses four domains: (1) patient selection—description of how patients were recruited such as inclusion and exclusion criteria; (2) index test—how the index test was conducted and interpreted; (3) reference standard—how the reference test was conducted and interpreted; and (4) flow and timing—patients that did not have the index or reference test or were excluded from final analysis. Each domain was assessed for risk of bias and the first three domains were also assessed for applicability and categorised as either low risk, high risk, or unclear. The index test was taken to be the intensity standardisation method under investigation, and the reference test was either no standardisation or an alternative method used as a comparator. Two reviewers (F.M., K.F.) independently reviewed each study and any disagreement resolved by consensus.

## Results

### Search results

**Fig. 2:**
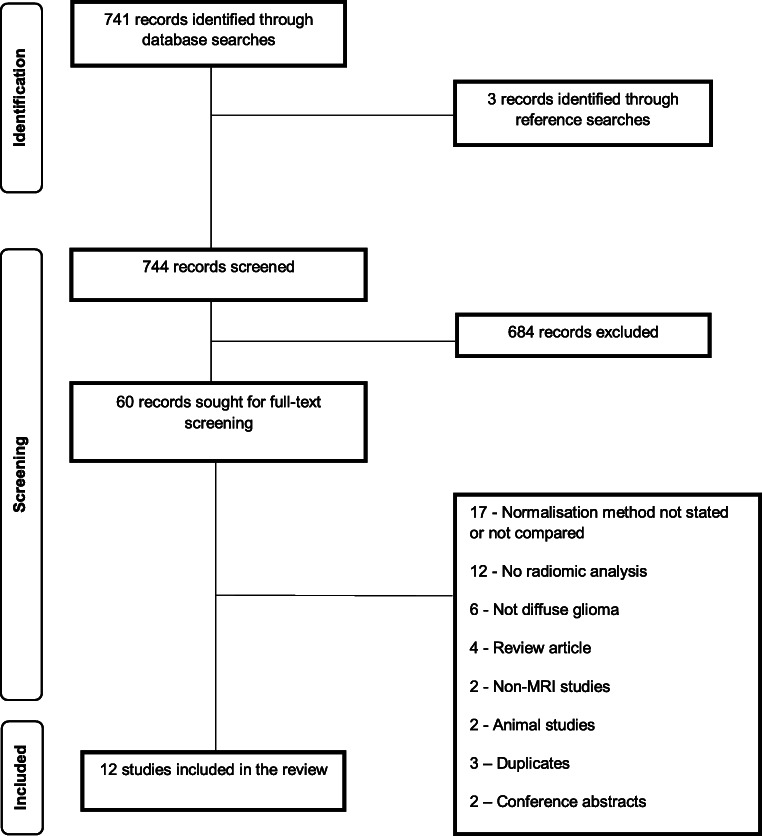
PRISMA flowchart illustrating the study selection for the systematic review of intensity normalisation in diffuse glioma radiomic studies

After duplicate removal, 741 results were returned from database searches (Fig. 2). Following title and abstract screening, full-text screening was undertaken for 60 articles. Twelve articles meeting the inclusion criteria were included in the review. Two studies by Florez et al [20, 21] were included separately as one used only radiomic features from a fluid-attenuated inversion recovery (FLAIR) sequence [21] and the other used a radiomics extracted from a combination of MRI sequences [20], and this may have an impact upon the results of any intensity standardisation process.

### Quality assessment

Risk of bias was assessed for each of the four domains and applicability assessed for the first three domains outlined above. Apart from risk of bias in the patient selection domain and applicability concern for the index test, all other domains were low risk for all studies (Table 1). Ten studies were deemed to have unclear risk due to lack of information on how patients were selected. It was unclear whether institutional patients were selected consecutively or randomly or, if publicly available datasets were used, it was unclear whether any inclusion/exclusion criteria were used to select patients.

**Table 1:** Summary of the risk of bias and applicability concerns for the 12 studies

Study	Risk of bias				Applicability concerns		
	Patient selection	Index test	Reference standard	Flow and timing	Patient selection	Index test	Reference standard
Chen et al 2019 [22]	Unclear	Low	Low	Low	Low	Low	Low
Zhao et al 2020 [23]	Unclear	Low	Low	Low	Low	Low	Low
Reuze et al 2018 [24]	Unclear	Low	Low	Low	Low	High	Low
Um et al 2019 [25]	Unclear	Low	Low	Low	Low	Low	Low
Upadhaya et al 2016 [26]	Unclear	Low	Low	Low	Low	High	Low
Florez et al 2018 [21]	Unclear	Low	Low	Low	Low	Low	Low
Florez et al 2018 [27]	Unclear	Low	Low	Low	Low	Low	Low
Hu et al 2021 [28]	Unclear	Low	Low	Low	Low	Low	Low
Hoebel et al 2021 [24]	Low	Low	Low	Low	Low	Low	Low
Vils et al 2021 [29]	Low	Low	Low	Low	Low	Low	Low
Carré et al 2020 [13]	Unclear	Low	Low	Low	Low	Low	Low
Orlhac et al 2020 [14]	Unclear	Low	Low	Low	Low	Low	Low

For applicability concerns of the index test, two studies [26, 27] were deemed high risk because it was not possible to isolate the effects of standardisation from other pre-processing. Two studies [24, 30] were low risk in all domains. Two studies by Florez et al [20, 21] also included patients with meningioma, but were not thought to be at risk of bias or an applicability concern as the results for the GBM patients were presented separately.

### Characteristics of included studies

Significant heterogeneity in the pre-processing steps and in analysis methodology (Table 2) precluded a meta-analysis and a narrative synthesis is presented.

**Table 2:** Summary of key features from the included studies (*n* = 12)

Study	Aims	Patients (train:test seta)	MRI sequences examined	Normalisation method	Pre-processing	Segmentation method	Radiomics software	Results	Conclusion
Chen et al 2019 [22]	To improve prediction of glioma grade using radiomics and the HSASR method of normalisation	521 (416:105)	T1Gd	HSASR method	Skull stripping and resampling	Manual	Pyradiomics	Highest AUC was 0.9934 for glioma grading with processing compared to 0.8512 without. The AUC after processing generally increased by more than 15%	Multicentre data processed by this method have good adaptability, which improves grading results and has value for clinical prediction
Zhao et al 2020 [23]	To examine the impact standardising MRI images with the HS-GS method has on using radiomics to predict glioma grades	693 (554:139)	T1Gd	HS-GS method	Skull stripping and resampling	Manual	Pyradiomics	The AUC of the predicted classification after HG-GS processing is 0.956 which is 26.96% higher than not performing a standardisation method	The results show that by adding HS-GS method to standard pre-processing, the diagnostic performance of using radiomics for glioma grading improves with respect to AUC, ACC, sensitivity, and specificity
Reuze et al 2018 [24]	To assess the effect of intensity rescaling on radiomic analysis of multicentre cohorts and the impact on the robustness of radiomic features	190 (n/a)	T1Gd	Intensity rescaling	Spatial resampling and discretisation of grey levels	Manual	LIFEx freeware	Out of the 31 textural features that were extracted, only 11 were deemed to be robust after the harmonisation method	Overall, the efficiency of the harmonisation method differed between devices, therefore it was not deemed to be a sufficient method to correct the differences between images
Um et al 2019 [25]	To determine the utility of a set of pre-processing methods on improving MRI radiomic feature robustness across multi-institutional datasets	161 (111:47)	FLAIR, T1W, and T1Gd	Histogram standardisation	Co-registration	Semi-automatic	Computational Environment for Radiotherapy Research (CERR)	From all of the pre-processing methods, histogram standardisation had a superior performance at reducing covariate shift, as Haralick, Soebel, and Laplacian of Gaussian features returned a significant decrease of Matthews correlation coefficient to 0.191, 0.170, and 0.140 respectively (*p* < 0.01)	From all the pre-processing methods, histogram standardisation contributes the most at the investigated measures such as feature dependence on scanner variability and covariate shift
Upadhaya et al 2016 [26]	To identify the impact of adding several pre-processing steps on the accuracy of the prognostic model which identifies patients above and below a median survival of 12 months	58 (58:58b)	T1W, T2W, T1Gd, and FLAIR	Dynamics intensity limitation	Bias field correction, skull stripping, co-registration, spatial resampling, and intensity quantisation	Automatic	Not identified	The additional pre-processing steps improved the prognostic model from sensitivity and specificity of 79% and 86% respectively to a sensitivity and specificity of 93%	The addition of investigated pre-processing methods highlights how various acquisition methods from different MR scanners can influence the accuracy of prognostic models
Florez et al 2018 [21]	To assess the ability of radiomic feature, to differentiate gross tumour volume (GTV) from oedema and differentiate vasogenic from tumour cell infiltration oedema	17 (17;n/a)	T1W, T1Gd, T2W, FLAIR and apparent diffusion coefficient (ADC)	1%-99% normalisation	Segmentation	Semi-automatic	MatLab version 2016a	Out of all of the sequences examined, T1Gd with 1–99% normalisation was the model best at classifying tumours with an AUC > 0.97	From the several hundred of radiomic feature extracted, only a small subset showed excellent ability to classify tumour tissue
Florez et al 2018 [27]	To assess the ability of radiomic features to distinguish oedema and infiltrative tumour based on FLAIR sequence	20 (20;n/a)	FLAIR	1–99% normalisation	Segmentation	Semi-automatic	MatLab version 2016a	Performance using single best discriminator reduced with addition of normalisation (AUC 0.87 vs 0.84) in patients with GBM	Small subset of texture features shows the ability to discriminate oedema from tumour
Hu et al 2021 [28]	To evaluate the impact MIL normalisation has on segmentation and feature extraction which allows the prediction of pathological grading and *IDH1* status	800 (533:267)	T1W, T1Gd, and FLAIR for all of the datasets (and T2W for the BraTs dataset, *n* = 285)	CycleGAN	Modality normalisation, layer spacing normalisation	Automatic	Not identified	MIL normalisation improved the AUC of pathological grading and IDH1 status prediction by 32% and 25% (*p* < 0.001) respectively. The accuracy of the pathological grading and IDH1 mutation prediction rose from 0.69 and 0.70 to 0.89 and 0.91 respectively after MIL normalisation	MIL normalisation can produce high-quality standardised data which is imperative for radiomic analysis
Hoebel et al 2021 [24]	To assess the impact of intensity normalisation methods (z-score normalisation and histogram matching) and intensity quantisation methods has on the repeatability and reproducibility of features extracted from a scan-rescan glioblastoma cohort.	48 (n/a)	T1Gd and FLAIR	*z*-Score normalisation and histogram matching	Segmentation, registration, bias field correction, and whole-brain extraction	Manual	Pyradiomics	For intensity features, both methods improved the repeatability on FLAIR images when compared to non-normalised baseline (*p* = 0.003 for *z*-score and *p* = 0.002 for histogram matching). This differs for T1Gd as both methods did not significantly effect the intraclass correlation coefficient of intensity features between scan and rescan	Both normalisation methods showed better repeatability for FLAIR images than T1Gd images, which may be a consequence of variations in contrast administration and timing of image acquisition after contrast administration
Vils et al 2021 [29]	To evaluate the association between radiomic features, clinical outcome, and molecular characteristic such as MGMT status	118 (69:49)	T1Gd	Linear intensity interpolation	Segmentation and manual extraction of brain tissue	Manual	Z-Rad	Regarding radiomic models capable of predicting MGMT status, images where the features were extracted from tumoural volumes of interest and normalised with linear interpolation were the only images validated in an independent cohort with an AUC of 0.670 (95% CI 0.5341–0.8056)	The proposed model may be a non-invasive approach to predict patient response to chemotherapy
Carré et al 2020 [13]	To assess the impact of three intensity normalisation methods coupled with grey level discretisation on the task of tumour grade classification in two independent cohorts	263 (195:48)	T1Gd and FLAIR	Nyul, WhiteStripe, and *Z* score normalisation method	Bias field correction, spatially resampled, skull-stripping, co-registration and segmentation	Manual	Pyradiomics	Significantly higher Jenson-Shannon divergence values were found on histogram and first-order features when comparing images with and without normalisation (*p* < 0.001 for Nyul, WhiteStripe and *Z*-score)	A combination of *z-*score normalisation and absolute discretisation produces the best results for models based on first and second order features
Orlhac et al 2020 [14]	To assess the impact of intensity normalisation and post-extraction realignment (ComBat) on the statistical distribution of radiomics from diffuse gliomas	18	T1Gd and FLAIR	Hybrid WhiteStripe (and ComBat)	Co-registration, bias field correction, spatial resampling	Manual	LIFEx freeware	69% of normal white matter, and 60% of tumour radiomics were significantly different following WhiteStripe (88 and 98% without WhiteStripe, respectively)	Intensity standardisation results in similar intensity values in images, but significant scanner-dependent changes require further correction with ComBat

*HSASR* histogram specification with automatic selection of reference, *HS-GS* histogram specification grid search

^a^Train/test numbers are only stated for any predictive model developed in the study; ‘n/a’ stated if no model was developed

^b^Model developed using leave one out cross-validation, according to stated references in the study

All studies were retrospective, although two studies [24, 30] utilised prospectively acquired data. Eight included multicentre data, and for one [27], it was unclear whether data comprised single or multicentre data. Five studies used a publicly available multicentre dataset from The Cancer Imaging Archive (TCIA) [29], or competition data from the brain tumour image segmentation benchmark (BraTs) [31] in addition to institutional data. One study [27] used only publicly available data.

The aims of the studies can be divided into two groups:

1. To assess the impact of intensity standardisation on the robustness and repeatability of radiomic features, and/or
2. To assess the impact of intensity standardisation on a predictive radiomics model.

Nine studies assessed the impact of intensity standardisation on a predictive model. Five studies assessed the impact of standardisation on feature robustness (two studies included both aims). Three groups, Hoebel et al [30], Carré et al [13], and Orlhac et al [14] used a ‘scan-rescan’ method to test radiomic feature robustness, which involved scanning the same patient after a short interval at different field strengths [13, 14] or on the same machine [30]. Two other studies, Um et al [32] and Reuze et al [26] assessed differences in the feature distribution between paired scanners or the ability of a classifier to distinguish patients scanned internally vs externally [32].

The three main approaches to intensity standardisation can be categorised as histogram matching, deep-learning, or limiting or rescaling the signal intensities. Most of the included studies evaluated one method; however, Carré et al [13] and Hoebel et al [30] used two or more. Further detail on the approaches is discussed in the upcoming sections.

### Histogram matching

Histogram matching involves transforming the signal intensities of an image to produce a match between the histogram of the reference and transformed image [25, 33]. The reference histogram is calculated from mean intensities of training images, at pre-specified intensity landmarks [33].

Um et al [32] assessed radiomic feature robustness after the following pre-processing steps: 8-bit rescaling, bias field correction, histogram matching, and isotropic resampling. A Random Forest classifier was used to predict whether images were from internal or external datasets and classification accuracy was measured using the Matthews correlation coefficient. A value of 1 means perfect prediction and 0 no better than chance, and therefore no scanner dependency. The value > 0.2 was taken to mean that images could still retain scanner dependence. Multiple classes of features were extracted. For edge features, different filters (Sobel, Laplacian of Gaussian, Gabor, wavelet) were applied and first-order features extracted. Haralick features were calculated from the grey-level co-occurrence matrices (GLCM). For baseline images, the Matthews correlation coefficients were 0.36, 0.22, and 0.39 (measured from the provided bar chart) for Haralick and the Sobel and Laplacian of Gaussian features, respectively. Histogram matching significantly decreased these to 0.191, 0.170, and 0.140 respectively (*p* < 0.01).

Zhao et al [34] used histogram specification-grid search (HS-GS), and Chen et al [23] used histogram specification with automated selection of reference frames (HSASR), which automatically select the training histogram. Zhao et al compared the predictive ability of standardised compared to unstandardised images for glioma grading demonstrating an area under the curve (AUC) of 0.956, 27% higher than that without standardisation. Using HSASR, Chen et al achieved 0.9934 AUC for grading (AUC 0.8512 without). These were the highest achieved for glioma grading, although a direct comparison to other methods of intensity standardisation would have been helpful in interpreting the results.

### Deep learning

Hu et al [22] describe ‘MIL’ pre-processing and intensity normalisation that corrects: modality incompleteness (M), uneven intensity distribution (I), and inconsistent layer spacing (L) in mpMRI datasets of T1-weighted (T1W), T1Gd, T2-weighted (T2W), and FLAIR sequences. Modality incompleteness is the absence of MRI sequences (referred to as ‘modalities’), for example T1Gd. Intensity unevenness is MRI signal intensity variation, and inconsistent layer spacing refers to variation in slice thickness. Effect of MIL normalisation on accuracy of radiomics model for glioma grading, for isocitrate dehydrogenase 1 (*IDH1*) prediction (a key genetic marker of adult-type diffuse glioma that has prognostic and diagnostic qualities), and on tumour segmentation was assessed. A cycle-consistent adversarial network (CycleGAN) standardised signal intensities, and a deep learning network synthesised any missing MRI sequences using an encoder (a modified U-net) and separate decoder [22]. Slice thickness was standardised using interpolation software, Statistical Parametric Mapping 12 (SPM12). AUC 0.693 (95% CI 0.613–0.772) was reported for unprocessed images, which increased following synthesis of missing sequences (AUC 0.838, 0.772–0.904), intensity standardisation (0.704, 0.626–0.783), and layer space normalisation (0.716, 0.639–0.793). Combining the three steps produced the best performing model (0.89, 0.838–0.941), highlighting the additive effects of the pre-processing pipeline.

### Limiting or rescaling signal intensity

Reuze et al rescaled the signal intensity between 0 and 32767 per patient and concurrently resampled to 0.5 × 0.5 × 0.5 mm^3^ and assessed the impact on feature robustness on images from 11 MRI scanners [26]. From 31 textural features, 11 were found to be robust among differing magnetic field strength post-normalisation (*p* > 0.05 on Wilcoxon paired test). Results from intensity standardisation alone were not presented.

Upadhaya et al assessed the effect of pre-processing steps on the accuracy of a overall survival (OS) prediction model [27]. Baseline pre-processing steps included bias field correction, skull stripping, and registration, with additional spatial resampling, intensity quantisation, and normalisation. Intensity normalisation ignored any values outside of the range: (*m-s*, *m+s*). *m* and *s* are the mean and standard deviation of the intensity values within the VOI. If the model utilised additional sequences and pre-processing steps, sensitivity improved from 79 to 93% and specificity from 86 to 93%. The effect of intensity standardisation alone was not presented.

Florez et al evaluated intensity standardisation on differentiation of tumour volume and oedema in 17 and 20 GBM patients [20, 21]. A 1–99% normalisation, where the 1^st^ and 99^th^ centiles of the intensity histogram are included [28], was compared to no normalisation. Normalised T1Gd sequences produced the best model with an AUC > 0.97 (0.85 without normalisation) [20]. The performance of normalised T2W images decreased—AUC of 0.85 (normalised) compared to AUC 0.91 (without). In a separate study, utilising only FLAIR, normalisation reduced AUC for discriminating tumour and oedema (AUC without 0.87, AUC with normalisation 0.84) [21].

Vils et al assessed the impact of linear intensity interpolation in 118 patients with recurrent GBM [24]. Linear intensity interpolation uses two regions of interests (ROIs) within normal contralateral white matter and the vitreous body:

$$ {intensity}_{normalized}={intensity}_{original}\frac{500}{intensity_{white\ matter}-{intensity}_{eye}\kern0.5em }+800-\kern0.5em \frac{500\ {intensity}_{white\ matter}}{intensity_{white\ matter}-{intensity}_{eye}\kern0.5em } $$

A radiomic model for prediction of O6-methylguanine-DNA methyltransferase (MGMT) promoter methylation (molecular marker for treatment response and prognostication) following normalisation achieved an AUC of 0.673 (95% CI 0.4837–0.8618) on the validation set. Without interpolation, the model achieved an AUC of 0.660 but could not be validated.

Orlhac et al assessed the impact of hybrid WhiteStripe normalisation on the distribution of features from normal white matter and tumours in 18 patients with diffuse glioma that had been scanned and rescanned at different field strengths [14]. WhiteStripe subtracts the mean and divides by the standard deviation of normal white matter intensity [35]. WhiteStripe reduced the number of significantly different features in normal white matter (88 to 69%) and tumour (98 to 60%), highlighting considerable remaining scanner dependency.

### Comparison of techniques

Carré et al [13] and Hoebel et al [30] both used histogram-matching and *Z*-score. *Z*-score normalisation subtracts the mean signal intensity from each voxel and divides by the standard deviation of the ROI [13]. Carré et al also used WhiteStripe.

Hoebel et al assessed the repeatability, using the intraclass correlation coefficient (ICC), of radiomic features extracted from a set of scan-rescan T1Gd and FLAIR images of 48 patients diagnosed with GBM [30]. *Z*-score and histogram matching improved repeatability of intensity features on FLAIR but not T1Gd. Histogram matching improved repeatability of texture features on FLAIR (*p* = 0.003), whereas *Z*-score did not and neither technique improved the repeatability of texture features on T1Gd.

Carré et al [13] assessed the impact of intensity normalisation on feature robustness and the prediction of glioma grading. Using a scan-rescan dataset of 20 patients with low-grade glioma, histogram matching was found to produce the highest number of robust first-order features on both T1Gd and FLAIR images (ICC and CCC > 0.80, 16 and 8 features out of 18 respectively). Regarding glioma grading using T1Gd images, and only robust features from the first scan-rescan experiment, the average balanced accuracy increased from 0.73 to 0.81, 0.79, and 0.81 for histogram, WhiteStripe, and *Z*-score respectively.

## Discussion

To be clinically useful, radiomics needs to be validated [36], with unique challenges when evaluating radiomic predictive models [37]. For MRI radiomics, a key challenge to assessing repeatability and reproducibility is to remove the scanner-dependent signal intensity changes [11]. This review confirms that intensity standardisation improves radiomic feature repeatability and improves most predictive models, and therefore that the clinical radiologist needs to be aware of this crucial step in any radiomics studies or applications. Variation in methodology precluded the direct comparison of results across studies and this review has highlighted potential areas of improvement, which may improve translation of radiomic models into the clinical setting (Table 3).

**Table 3:** Limitations of the current literature and opportunities for the future

Limitation	Opportunity
1. Assessing the effect of multiple preprocessing steps simultaneously	Effects of preprocessing steps presented independently of others so their effect on the result can be determined
2. Investigating the effect of only one intensity standardisation technique	Impact of more than one standardisation method on a predictive model or feature robustness should be evaluated
3. Lack of scan-rescan data used to test the repeatability of radiomic features	Increased availability of datasets that have rescanned a patient with a diffuse glioma within a short time interval (i.e. days) in public databases
4. Single-centre studies used to assess standardisation techniques	Use of multi-centre datasets in assessing the efficacy of standardisation techniques and repeatability of radiomic features

In two studies [26, 27], the effects of intensity standardisation were difficult to differentiate from other pre-processing, and the authors could have reported separately the impact of different pre-processing steps on feature robustness or model performance. Hu et al presented all possible combinations of pre-processing steps, with separate AUC results, so the impact of each step was identifiable.

Only two studies [13, 30] compared more than one intensity technique. Given the number of methods and lack of consensus, more studies that directly compare techniques are required. This is important when interpreting the results of histogram specification studies [23, 34]. The AUC for grading was the highest reported; however, it is unclear how this relates to other techniques. A recent analysis [16] compared multiple intensity standardisation techniques and post-feature extraction correction with ComBat, a statistical normalisation for batch-effect correction in genomics that has been applied to radiomics [11, 14]. Intensity standardisation was insufficient to remove scanner dependency, but ComBat could remove scanner-dependent information from extracted features [16], similar to the findings of Orlhac et al [14].

Three studies used scan-rescan data, providing the opportunity to assess radiomic feature reproducibility on images from the same patient acquired within a short time delay (i.e. days between studies). Although a tumour may change microscopically within several days, these radiomic studies assume that if the imaging appearance remains the same then the radiomic features ought to as well [13, 14, 30]. Test-retest data, along with phantom studies [16], and comparison of radiomic features extracted from normal structures provide a useful paradigm to test standardisation techniques. Open access to such data in a public repository should help further validate different intensity standardisation approaches.

Limitations to this review include not being able to retrieve full-text articles for two conference abstracts. Based on the abstracts, it is unlikely they would have been included. Their potential omission will have had a limited impact as a narrative synthesis would still have been required. QUADAS-2 is not specifically designed for assessing the efficacy of MRI intensity standardisation techniques, but we considered this a viable method given the absence of a more specific alternative. The scope of this review was to assess MRI intensity standardisation in the context of diffuse glioma and there will have been the inevitable omission of studies of other organs, brain pathologies, and healthy volunteers.

## Conclusion

No clear consensus has emerged as to which approach is the most reliable standardisation approach. In order to translate radiomics to the clinic, studies should assess the effects of intensity standardisation on their results and the impact of any intensity standardisation step should be clearly reported. Collation and sharing of scan-rescan datasets would facilitate production of radiomic models in diffuse glioma and greatly improve the development of clinically translatable models.

## Supplementary Information

ESM 1 (DOCX 27 kb)

ESM 2 (DOCX 35 kb)

ESM 3 (DOCX 31 kb)

## Abbreviations

BraTs: Brain tumour image segmentation benchmark
CycleGAN: Cycle-consistent adversarial network
FLAIR: Fluid-attenuated inversion recovery
GBM: Glioblastoma
GLCM: Grey-level co-occurrence matrices
HSASR: Histogram specification with automated selection of reference frames
HS-GS: Histogram specification-grid search
ICC: Intraclass correlation coefficient
IDH1: Isocitrate dehydrogenase 1
MGMT: - O6-methylguanine-DNA methyltransferase
mpMRI: Multiparametric MRI
OS: Overall survival
PRISMA: Preferred Reporting Items for Systematic Reviews and Meta-Analysis
QUADAS-2: Quality Assessment of Diagnostic Accuracy Studies 2
ROI: Region of interest
SPM12: Statistical Parametric Mapping 12
T1Gd: T1-weighted gadolinium enhanced
T1W: T1-weighted
T2W: T2-weighted
TCIA: The Cancer Imaging Archive
VOI: Volume of interest

## Declarations

### Guarantor

The scientific guarantor of this publication is SC.

### Statistics and biometry

No complex statistical methods were necessary for this paper.

### Methodology

• retrospective

• observational

• performed at one institution
