# Supplementary material for: Intensity standardization of MRI prior to radiomic feature extraction for artificial intelligence research in glioma—a systematic review
Source: Eur Radiol. 2022 Apr 29;32(10):7014–25. doi: 10.1007/s00330-022-08807-2 (PMC9474349; doi:10.1007/s00330-022-08807-2)
Supplement: Supplementary file 1 — (DOCX 27 kb) [file 330_2022_8807_MOESM1_ESM.docx]

**Supplementary information**

**Materials and methods**

Search strategy and selection criteria

A search of MEDLINE, EMBASE and SCOPUS databases was performed on October 5th 2021 using the search strategy outlined below. Records were managed using citation management software, and automatic duplicate removal was used to screen the results. Two reviewers (KF, FM) independently and manually reviewed the titles and abstracts and subsequently the full texts to determine if they satisfied the inclusion and exclusion criteria. Any disagreement was resolved by consensus.

Inclusion criteria:

- Original research article
- Adult (≥16 years old) patients with diagnosis of adult-type diffuse glioma included
- Application of intensity normalisation method to imaging prior to extraction of radiomic features
- Comparison of the effect of standardisation to either no standardisation or another standardisation method
- Radiomic features extracted from images

Exclusion criteria:

- Non-human based study
- No patients with adult-type diffuse glioma
- Non-original research (ie. review article)
- Non-MR radiomics extracted
- No mention of intensity standardisation
- No assessment of the effect of intensity standardisation (comparison with another method of standardisation

The full-text of included studies were read by both of the two reviewers (FM, KF). They worked in conjunction to extract relevant data that was tabulated and presented in the results section, including:

- First author,
- Publication year
- Study aims
- Number of participants
- Division of patients into training and test cohorts (if applicable)
- MRI sequences used
- Method of intensity standardisation
- Any pre-processing steps applied prior to radiomic extraction
- Radiomic extraction software
- Main result
- Study conclusion

Outcomes and data-extraction

The primary outcome for which data were sought was the efficacy of intensity standardisation methods. The measurement of standardisation efficacy was not restricted to any statistic or method due to lack of an agreed standard. It was not known how varied the studies would be in their design and reporting. If studies developed a predictive or diagnostic model, the AUC of was extracted. For any studies that did not develop a predictive model, the primary outcome effect was taken as reported efficacy of intensity standardisation in the study. All effect measures were agreed by consensus between three reviewers (KF, FM, SC).

Meta-analysis was precluded by heterogeneity of the included studies and therefore a narrative synthesis was presented. In the narrative synthesis, the method of intensity standardisation was used to group studies without any overlap between groups. No preparation or processing of data within the included studies was undertaken – results of studies were included as they appeared in published manuscripts.

Since a meta-analysis was not conducted, additional methods such as sensitivity analyses or subgroup analyses were not performed. Similarly, the impact of missing results was not relevant as we included any outcome measure presented in the studies. No additional methods were used to assess certainty or confidence in the outcome, other than qualitative assessment of each study during the narrative synthesis.

This systematic review was not prospectively registered on a registry.

Search strategies:

The following search strategy was used to search Medline and then EMBASE, both of which were accessed via OVID on 05/10/2021. No limits or filters were applied.

1. MRI.ab,sh,ti.

2. magnetic resonance imaging.ab,sh,ti.

3. 1 or 2

4. AI.ab,ti.

5. Machine learning.ab,sh,ti.

6. Neural network.ab,sh,ti.

7. "Neural network*".ab,ti.

8. "Radiomic*".ab,ti.

9. "radiogenomic*".ab,ti.

10. deep learning.ab,ti.

11. Advanced neuroimaging.ab,ti.

12. Artificial intelligence.ab,sh,ti.

13. 4 or 5 or 6 or 7 or 8 or 9 or 10 or 11 or 12

14. "intensity standard*".ab,ti.

15. "intensity harmon*".ab,ti.

16. (intensity adj10 standard*).ab,ti.

17. (intensity adj10 harmon*).ab,ti.

18. "image preprocess*".ab,ti.

19. feature extraction.ab,ti.

20. extracted feature.ab,ti.

21. radiomic feature.ab,ti.

22. texture feature extraction.ab,ti.

23. "harmon*".ab,ti.

24. "standard*".ab,ti.

25. 14 or 15 or 16 or 17 or 18 or 19 or 20 or 21 or 22 or 23 or 24

26. Glioma.ab,sh,ti.

27. Glioblastoma.ab,sh,ti.

28. glioblastoma multiforme.ab,sh,ti.

29. GBM.ab,ti.

30. Low grade.ab,ti.

31. Grade II.ab,ti.

32. 30 or 31

33. 26 and 32

34. High grade.ab,ti.

35. Grade III.ab,ti.

36. Grade IV.ab,ti.

37. 34 or 35 or 36

38. 26 and 37

39. "Glial cell tumo*".ab,ti.

40. "Astrocytoma*".ab,ti.

41. "Oligodendroglioma*".ab,ti.

42. "Oligoastrocytoma*".ab,ti.

43. brain cancer.ab,ti.

44. Neuro-oncology.ab,ti.

45. 26 or 27 or 28 or 29 or 33 or 38 or 39 or 40 or 41 or 42 or 43 or 44

46. "normali*ation".ab,ti.

47. 14 or 15 or 16 or 17 or 18 or 19 or 20 or 21 or 22 or 23 or 24 or 46

48. 3 and 13 and 45 and 47

The following strategy was used to search Scopus on 05/10/21. No limits or filters were applied.

( TITLE-ABS ( mri )  OR  TITLE-ABS ( magnetic  AND  resonance  AND  imaging ) )

AND

( TITLE-ABS ( artificial  AND  intelligence )  OR  TITLE-ABS ( ai )  OR  TITLE-ABS ( machine  AND  learning )  OR  TITLE-ABS ( neural  AND  network )  OR  TITLE-ABS ( radiomic* )  OR  TITLE-ABS ( radiogenomic* )  OR  TITLE-ABS ( deep  AND  learning )  OR  TITLE-ABS ( advanced  AND  neuro-imaging ) )  AND  ( TITLE-ABS-KEY ( intensity  AND  standard* )  OR  TITLE-ABS-KEY ( intensity  AND  harmon* )  OR  TITLE-ABS ( intensity  AND  adj10  AND  standard* )  OR  TITLE-ABS ( intensity  AND  adj10  AND  harmon* )  OR  TITLE-ABS-KEY ( image  AND  preprocess* )  OR  TITLE-ABS ( feature  AND  extraction )  OR  TITLE-ABS ( extracted  AND  feature )  OR  TITLE-ABS ( radiomic  AND  feature )  OR  TITLE-ABS ( texture  AND  feature  AND  extraction )  OR  TITLE-ABS-KEY ( harmon* )  OR  TITLE-ABS ( standard* )  OR  TITLE-ABS-KEY ( normali*ation ) )

AND

( TITLE-ABS ( glioma )  OR  TITLE-ABS ( glioblastoma )  OR  TITLE-ABS ( glioblastoma  AND  multiforme )  OR  TITLE-ABS ( gbm )  OR  TITLE-ABS ( low  AND  grade  AND  glioma )  OR  TITLE-ABS ( grade  AND  ii  AND  glioma )  OR  TITLE-ABS ( high  AND  grade  AND  glioma )  OR  TITLE-ABS ( grade  AND  iii  AND  glioma )  OR  TITLE-ABS ( grade  AND  iv  AND  glioma )  OR  TITLE-ABS ( glial  AND  cell  AND  tumo* )  OR  TITLE-ABS ( astrocytoma* )  OR  TITLE-ABS ( oligodendroglioma* )  OR  TITLE-ABS ( brain  AND  cancer )  OR  TITLE-ABS ( neuro-oncology ) )
